# Ethnic Friendship Networks of Korean-American Older Adults in Subsidized Senior Housing

**DOI:** 10.1093/geroni/igaf122.2990

**Published:** 2025-12-31

**Authors:** Kyungmin Kim, Juyoung Park, Yuri Jang

**Affiliations:** Seoul National University, Seoul, Republic of Korea; Emory University, Georgia, United States; University of Southern California, Los Angeles, California, United States

## Abstract

Senior housing communities are an important social environment for older adults, offering opportunities for interactions with peers and a sense of community. Older adults from ethnic minorities often find their ethnic peers within housing communities as a primary source of social and emotional support. This study examined ethnic friendship networks that older immigrants experience in senior housing and related individual characteristics. This study analyzed data from Korean-American older adults who reside in subsidized senior housing in the Greater Los Angeles Area (person N = 342; aged 65–102; facility N = 6). Participants named ethnic friends within their housing facilities up to three, using a name-generator approach of social network analysis. Poisson regression models were estimated to examine participants’ characteristics associated with degree centrality of ethnic friendship network: (a) out-degree (i.e., self-reported friendship with other residents) and (b) in-degree (i.e., nominated friendship from other residents). Out-degree centrality was positively associated with female gender, participation in community activities, social engagement, and years of residence in the facility. In-degree centrality was positively associated with participation in community activities, chronic medical conditions, and social interactions in the facility. We also found that both indicators of degree centrality substantially differed across six housing facilities, suggesting that each facility may serve as unique social and built environments for ethnic friendship networks of residents. Our findings underscore identifying different levels of factors contributing to ethnic friendship in the senior housing community, given the benefits of being part of a friendship network in later life.

